# Taxonomic Distinctness and Richness of Helminth Parasite Assemblages of Freshwater Fishes in Mexican Hydrological Basins

**DOI:** 10.1371/journal.pone.0074419

**Published:** 2013-09-27

**Authors:** Benjamín Quiroz-Martínez, Guillermo Salgado-Maldonado

**Affiliations:** Universidad Nacional Autónoma de México, Instituto de Biología, Laboratorio de Helmintología, México D. F. México; Institut national de la santé et de la recherche médicale - Institut Cochin, France

## Abstract

In this paper, we analyse the distributional patterns of adult helminth parasites of freshwater fishes with respect to the main hydrological basins of Mexico. We use the taxonomic distinctness and the variation in taxonomic distinctness to explore patterns of parasite diversity and how these patterns change between zoogeographical regions. We address questions about the factors that determine the variation of observed diversity of helminths between basins. We also investigate patterns of richness, taxonomic distinctness and distance decay of similarity amongst basins. Our analyses suggest that the evolution of the fauna of helminth parasites in Mexico is mostly dominated by independent host colonization events and that intra - host speciation could be a minor factor explaining the origin of this diversity. This paper points out a clear separation between the helminth faunas of northern - nearctic and southern - neotropical components in Mexican continental waters, suggesting the availability of two distinct taxonomic pools of parasites in Mexican drainage basins. Data identifies Mexican drainage basins as unities inhabited by freshwater fishes, hosting a mixture of neotropical and nearctic species, in addition, data confirms neotropical and neartic basins/helminth faunas. The neotropical basins of Mexico are host to a richest and more diversified helminth fauna, including more families, genera and species, compared to the less rich and less diverse helminth fauna in the nearctic basins. The present analysis confirms distance - decay as one of the important factors contributing to the patterns of diversity observed. The hypothesis that helminth diversity could be explained by the ichthyological diversity of the basin received no support from present analysis.

## Introduction

The fauna of helminth parasites of freshwater fishes of Mexico is one of the best known in Latin America [1], with more than 260 helminth species recorded [2], the knowledge of helminthofauna that parasitizes freshwater fishes of Mexico has greatly increased in recent times [2], [3], [4]. Recently, through the analysis of the taxonomic composition and richness of helminth species of fish in 23 drainage basins from Mexico, Quiroz-Martínez & Salgado-Maldonado [5] showed that basins can be characterised by the helminth fauna parasitizing freshwater fishes, in the same way as the hosts themselves characterise these basins. This analysis helped identify the variation in helminth diversity between basins of Mexico; different drainage basins harbour different numbers of parasite species, though the reasons why have yet to be examined. Our analysis pointed out a rich set of neotropical species in the neotropical basins [5], while nearctic basins host a less rich set of nearctic helminth species. Long held assumptions about Mexican biogeography are that the biota is divided into northern and southern components, but regarding the helminth parasites of freshwater fishes, this pattern still represents a hypothesis to be tested, as new information on species distribution is being accumulated.

In addition, our previous analyses [5] suggest that the diversity of helminths in a basin is related to its fish host diversity. There are families of fish with high richness of helminths, while other families are less parasitized [3], this explains why basins that host fish families rich in parasites or that host more fish families are the richest basins in terms of helminth parasites.

To date, a number of studies have used species richness as a measure of diversity for helminth assemblages. However, diversity is a complex concept, much more inclusive than just the total number of species in a given area [6], [7]. Species richness does not capture all facets of diversity [6] as it ignores, for example, the evolutionary relationships among species coexisting in an assemblage [8]. The diversity of parasites in an assemblage can be increased by intra-host parasite speciation events or by host-switching windows [9]. The inability to distinguish between these possible evolutionary origins of parasite diversity is a limitation when using richness as a measure of diversity [10]. The use of diversity measures other than species richness can cast a different light on certain aspects of biodiversity. Recently, the taxonomic relationships among species have been used to describe another facet of biodiversity. Several recent papers have examined the appropriateness of taxonomic distinctness as an alternative measure of parasite diversity in comparative studies [11], [12], [13], [14]. The taxonomic distinctness index is a measure of diversity that incorporates information on the relationships among species of parasite assemblages in an attempt to recognise diversification processes; it seeks to capture phylogenetic diversity and is closely linked to functional diversity; it captures both, the distribution of abundances amongst species and the taxonomic relatedness of the species in each sample [15]. Taxonomic distinctness quantifies diversity as the relatedness of the species within a sample, based on the distances between species in a classification tree [7], [16] and it is a measure of the average degree to which individual species in an assemblage are related to each other. The average taxonomic distinctness (AvTD), is the mean of all distances between all species through the taxonomic tree, for all pairs of species within a sample, and it measures the taxonomic breadth of the sample [7].

Furthermore, Clarke & Warwick [15] developed another diversity index, VarTD or variation in taxonomic distinctness. It is calculated as the variance of pairwise path lengths used to calculate the taxonomic distinctness. This means that VarTD is the variation in branch lengths amongst all pairs of species. This index is a measure of the irregularities and divergences in the distribution of branch lengths within a sample [7]. Both indices are interesting because, in their simplest form, they can be calculated from presence/absence data, neither is affected by the number of species or the sampling effort, and they appear relatively insensitive to major habitat differences [10], [17], [18]. These new measures of diversity will undoubtedly bring a better understanding of how parasite biodiversity evolves [19].

Taxonomic distinctness focuses on evolutionary relationships among species coexisting in an assemblage and represents the average evolutionary distance among all species. Poulin and Mouillot [10] used this index for endoparasite species of mammalian hosts in order to explore host features as factors in the structure of parasite assemblages. They were able to show that the taxonomic distinctness of a host’s helminth assemblage is similar enough across conspecific host populations to be considered a host specific trait [10]. Luque et al. [12] used taxonomic distinctness to assess parasite diversity in marine fishes from Brazil. Although on a local scale, these authors showed this index revealed patterns different from those based on parasite species richness, and that shifting the focus from species richness to taxonomic diversity could cast a different light on the evolution of fish parasite biodiversity.

In this study, we use the taxonomic distinctness and the variation in taxonomic distinctness in order to address questions about the factors that determine the variation of the observed diversity of helminth parasites of freshwater fishes between drainage basins of Mexico. We use both taxonomic distinctness and variation in taxonomic distinctness to provide a robust summary of taxonomic relatedness patterns of helminth parasites. We explore patterns of parasite diversity observed between biogeographic regions of Mexico seeking patterns of diversity amongst 23 Mexican major hydrological basins, using the adult helminth parasites of freshwater fishes to typify each basin. This is done in order to examine how patterns change between zoogeographical regions as well as to investigate patterns of taxonomic distinctness and distance decay of similarity amongst basins. Specifically, we try to test the hypothesis that variability in the observed diversity is correlated with fish diversity, that basins richest in families and genera of fish are the most diverse in terms of parasites. Among the factors that might contribute to explain the ichthyological diversity of a basin, we can consider structural characteristics such as the diversity of habitats suitable for fish hosts and their isolation from other basins. Basins with greater diversity of environments, and closer to each other, will have high fish diversity. By contrast, the isolation of a basin prevents colonisation by additional groups of fish; i.e. the geographical distance amongst basins can be an important determinant of the pattern of distribution of the helminths. We therefore expect that drainage basins hosting more fish families, genera, and species, would be, in general, those with most diverse helminth assemblages. Conversely, the more isolated basins would be the less rich in terms of fish assemblages and as a consequence these basins would have the less diverse helminth assemblages.

## Materials and Methods

**Figure 1:**
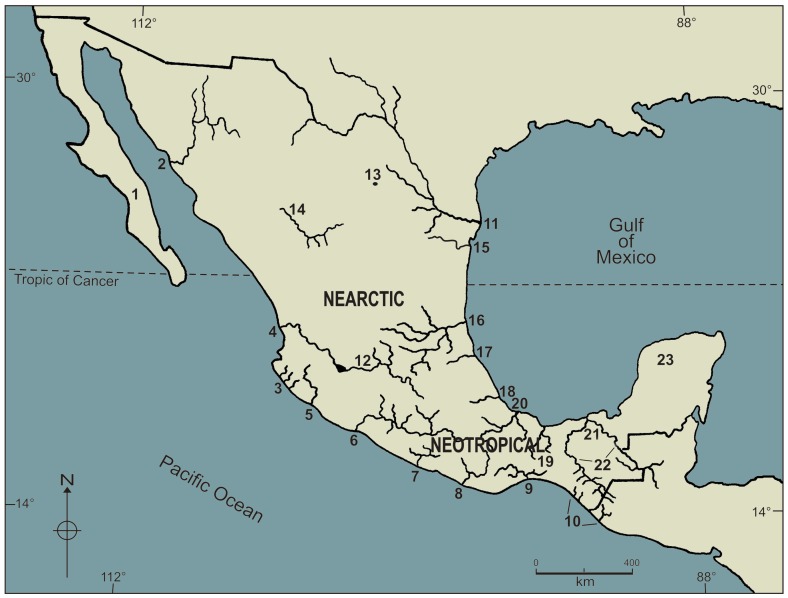
Mexican hydrological features. The code used to identify each basin is: 1, Oases of Baja California Sur; 2, Río Yaqui; 3, rivers near Chamela, Jalisco; 4, Río Santiago; 5, Río Armería-Ayuquila; 6, Río Balsas; 7, bodies of water in Guerrero, including Río Papagayo; 8, Río Atoyac; 9, Río Tehuantepec; 10, rivers along the south Pacific coast of Chiapas; 11, Río Bravo; 12, Río Lerma; 13, bodies of water of the Valley of Cuatro Ciénegas; 14, Río Mezquital, Río Nazas and springs of Durango; 15, Río San Fernando, Río Soto La Marina and other bodies of water in Tamaulipas; 16, Río Pánuco; 17, Río Tuxpan; 18, Río La Antigua; 19, bodies of water of Los Chimalapas; 20, Río Papaloapan; 21, bodies of water in coastal plain of Tabasco; 22, basins of Río Usumacinta and Río Grijalva, Chiapas; 23, bodies of water of the Yucatán Península.

We revisited the database already published by Salgado-Maldonado & Quiroz-Martínez [3]. This database includes data for every adult helminth species recorded to date from freshwater fishes of Mexico; it consists of a binary matrix where species’ presence/absence in 23 drainage basins from Mexico was marked as 1 or 0, respectively. Location of the basins and the code used to identify each in the subsequent plots are shown in Fig. 1. The information included in the initial matrix was subsequently aggregated into the corresponding supra-generic levels, such that for every species it includes the relationships to genus, family, class, and phylum, updated from the previous taxonomical scheme available from Salgado-Maldonado [2]. We calculated two indices to compare the parasite diversity of the various basins: 1) the Average taxonomic distinctness (AvTD, Δ+), and 2) the Variation in taxonomic distinctness (VarTD, Λ+), as defined by Warwick & Clarke [11] and Clarke & Warwick [20].

The Average taxonomic distinctness (AvTD, Δ+) was calculated using the next function:
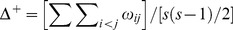
(1)where ω*ij* is the phylogenetic/taxonomic path length between species *i* and *j*, and *s* is the number of species).

The Variation in taxonomic distinctness (VarTD, Λ+), was calculated as:
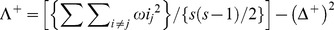
(2)


The Average taxonomic distinctness (Δ+) of parasite species measures the average taxonomic distance between different parasite species in an assemblage; the greater the value of Δ+, the greater the average taxonomic difference between species in the assemblage [13]. VarTD is the variation in branch lengths amongst all pairs of species and measures the distribution of branch lengths within a sample; the greater the variation in branch lengths, the greater the value of Λ+. The computation of the indices from the taxonomic hierarchy of the parasite species based on the Linnaean classification into phyla, classes, families, genera and species was made using the Plymouth Routines in Multivariate Ecological Research PRIMER v6 [16], [21]. The resulting matrices were examined to derive dissimilarity patterns by means of cluster analysis (group average linkage) and non-metric multidimensional scaling (nMDS), as suggested by Field et al. [22] and Clarke & Warwick [16]. The cluster and MDS analyses were run on the θ^+^ taxonomic dissimilarity index, a presence/absence “beta diversity” coefficient [16], [23].

To identify differences in taxonomic distinctness (AvTD, Δ^+^), and in variation in taxonomic distinctness (VarTD, Λ^+^), from expected Δ+ or Λ+ values derived from the total species list of helminth parasites of freshwater fishes of Mexico, we performed a randomization procedure (as suggested by Clarke & Warwick [20], [24] and by Warwick & Clarke [25]) for any observed set of species for Mexican river basins, using the inventory of helminth species recorded in Mexico [3], constructed from individual lists of species recorded in each basin. This procedure defines a theoretical model of the regional context of the taxonomic composition of helminth parasites of freshwater fishes of Mexico. A simulated distribution was developed leading to a theoretical mean (an horizontal line displaying the taxonomic distinctness for all helminth parasites of freshwater fishes of Mexico shown in the resulting Δ^+^ and Λ^+^graphs ) and to a confidence funnel for each, Δ^+^, and Λ^+^, from random subsamples of helminth species of freshwater fish in Mexico [20], [24], [25], [26]. The simulation generates a large number of random subsets of species from the total Mexican species list, each of size *m*, computes the corresponding Δ^+^ and Λ^+^ values, and determines an interval in which 95% of these values lie. Values of AvTD and VarTD located within the 95% probability funnel indicate that species diversity in the corresponding areas falls within the expected range, thus allowing both for sample size, and sample effort free diversity comparisons.

The relationship between the number of genera (G) and the number of species (S) of helminth parasites in freshwater fishes from all drainage basins used in this study was examined by correlation and graphic methods. In order to evaluate the effects of geographical distance on species composition (Distance Decay) we calculated the distance in km between all pairs of basins and plotted the calculated taxonomic distinctness Δ^+^, as a function of distance using a linear function to fit the scatter plot. We used the geographical centre of the basin or a sampled point near the geographical centre as a point of reference to calculate the distance between all pairs of basins studied. Finally, we explored the relationship between diversity (measured both as species richness and as Δ^+^ of helminth parasites freshwater fish through 23 Mexican basins and the ichthyological diversity (Numbers of families, genera and species of fish each basin harbour taken from Miller et al. [27]). These analyses were made using Matlab software.

## Results

Our revisited database includes a total of 180 species of adult helminths from 84 genera and 34 families belonging to three different phyla (Platyhelminthes, Acanthocephala and Nematoda), recorded from 17 families of freshwater species of fishes from 23 drainage basins across Mexico. This database is already available from Salgado-Maldonado and Quiroz-Martínez [3] and has been previously described and examined [3], [5].

**Figure 2:**
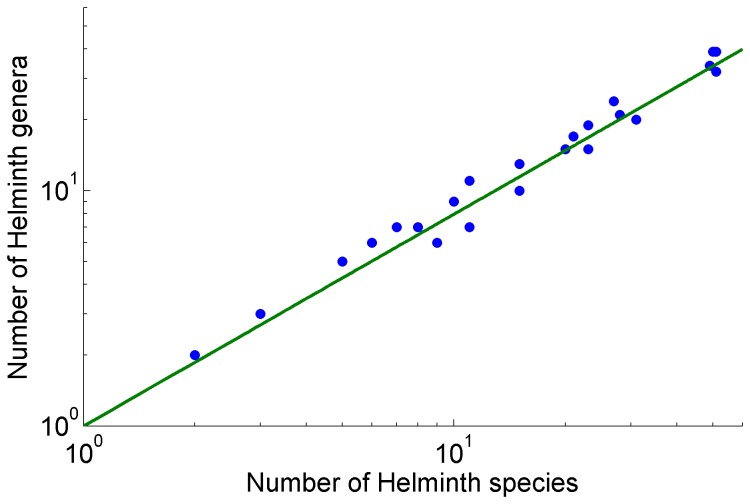
Relationship between the number of genera (G) against the number of species (S) across 180 species helminth parasites of freshwater fishes of Mexico.

The relationship between the number of helminth genera and the number of helminth species was linear and positive (Fig. 2), and the number of genera represented in this assemblage increases as a power function of the number of species. The exponent of the power function (0.9) is almost one, suggesting that almost all parasite species belong to different genera. Further exploration of the database showed that only 18%, 15 of the 84 genera have more than two species (Table S1).

**Figure 3:**
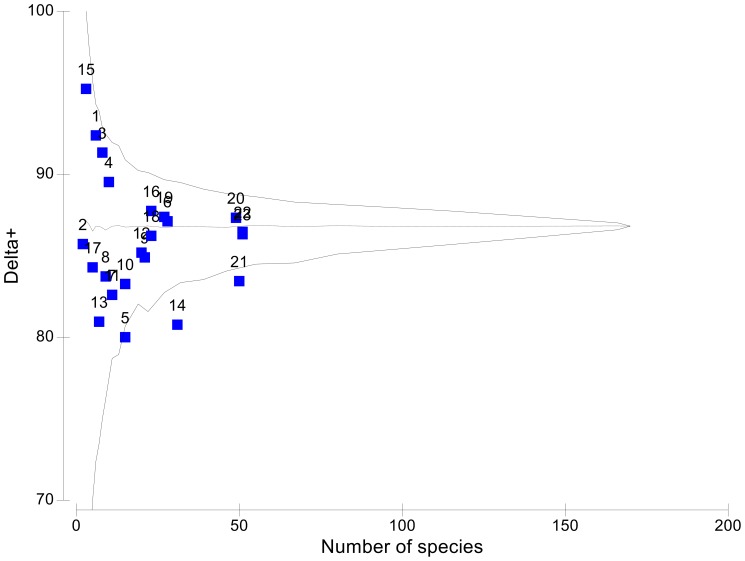
Simulated distribution of average taxonomic distinctness (theoretical mean, horizontal, dashed line) for random subsets of species from the full species list of 180 helminth species from freshwater fishes of Mexico, and the 95% confidence limits (funnel) of taxonomic distinctness, Δ^+^. Superimposed to the theoretical model are shown the actual values of Δ^+^ for each of 23 helminth species list from Mexican hydrological basins plotted against the number of species in each basin.

The estimated Δ^+^ values resulting from the helminth species list in each of the 23 Mexican freshwater basins, plotted against the number of species in each basin, are shown in Fig. 3, superimposed on the 95% funnel for the simulated distribution of Δ+ for subsets of fixed number of species drawn randomly from the 180 helminth species of freshwater fishes of Mexico. Most of the calculated Δ+ values were close to the expected simulate mode of the funnel. According to these Δ+ values, most of the Mexican basins are as diverse as expected, because they are located inside of the funnel. However, Tabasco (21) and Durango (14) basins are below the lower limit of the simulated distribution under the null hypothesis. Rather unrealistic samples available up to date from Río Yaqui (2), Río San Fernando, Soto La Marina and other bodies of water of Tamaulipas (15), and Río Tuxpan (17), preclude the usage of these basins for this part of analysis of diversity (see data on Table S2).

The Oases of Baja California (1), rivers close to Chamela (3), and Río Santiago (4) ranked amongst the most diverse basins (Fig. 3); high values of Δ+ show they are less similar to each other, as they have a high number of higher taxonomic categories. These three basins have few species (S = 4 to 8) but their records include the three higher taxa, phyla Platyhelminthes, Acanthocephala and Nematoda, with an noticeable evenness accounting for the distribution of genera and species in classes and phyla (Table S2). Conversely, lower Δ+ values showing less taxonomical diversity, were recorded from the richest (S = 49–51) basins, Papaloapan (20), Tabasco (21), Usumacinta (22), and Yucatán Peninsula (23). All three phyla and respective classes have been recorded from these basins (Table S2, Additional materials); however, the distribution from lower to higher taxa is less even, displaying a clear dominance by trematodes, nematodes and monogeneans, and a comparatively reduced numbers of acanthocephalans and cestodes. Between these two extremes all other basins (Fig. 3) ranked with comparatively medium Δ+ diversity values, and medium richness levels (S = 20–28). The less diverse basins, also have a limited richness (S = 9–15). Some phyla or classes are absent from these river basins. For example, Río Atoyac (8), and Río Bravo (11) basins lack records of acanthocephalans; on the other hand, Guerrero (7), Ayuquila (5), and the rivers along the Pacific coast of Chiapas (10), lack records of either monogeneans or cestodes, or both (see Table S2).

**Figure 4:**
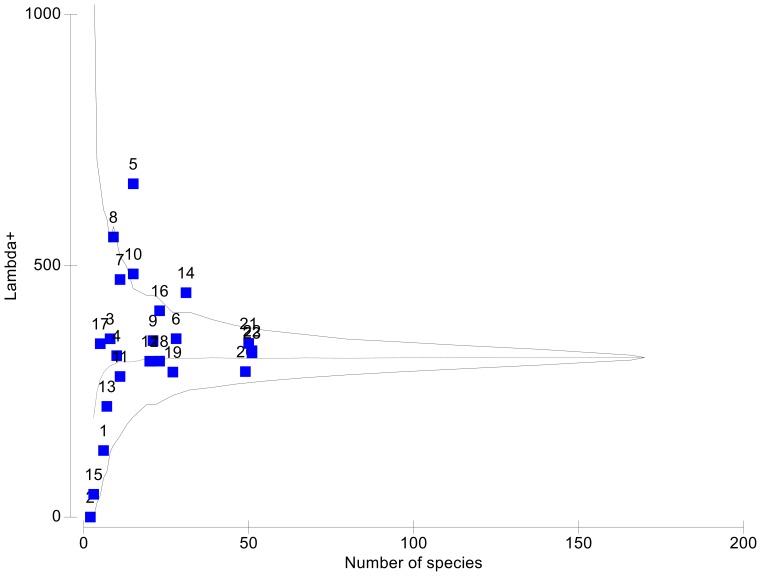
Simulated distribution of the variation in taxonomic distinctness (theoretical mean, horizontal, dashed line) for random subsets of species from the full species list of 180 helminth species from freshwater fishes of Mexico, and the 95% confidence limits (funnel) of taxonomic distinctness, Λ^+^. Superimposed to the theoretical model are shown the actual values of Λ^+^ for each of 23 helminth species list from Mexican hydrological basins plotted against the number of species in each basin.

**Figure 5:**
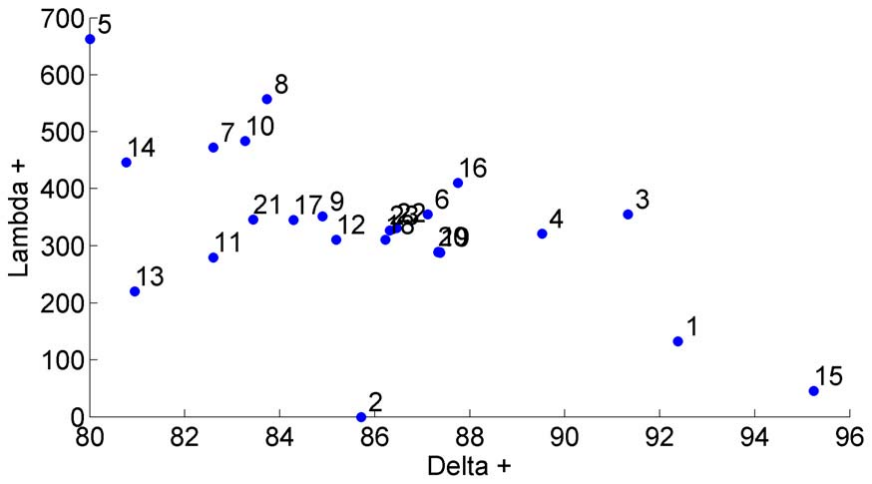
Relationships between taxonomic distinctness Δ^+^ and variation in taxonomic distinctness Λ^+^. Values were calculated for all 23 drainage basins used in this study.

The same pattern already described for Δ+ values, is confirmed by trends observed for Λ+ values (Fig. 4). Most of the calculated Λ+ values were close to the simulated mode of the funnel, or even higher, falling beyond the upper limit of the simulated distribution. Combining both measures of diversity, Δ+ and Λ+, suggests that in the Ayuquila (5), Durango (14), Atoyac (8), and Chiapas Pacífico (10) river basins, there are more closely related species than in other basins; Δ+ values ranked rather low (Fig. 3), while Λ+ values ranked high in these four basins (Fig. 4). A closer look at the records from these four basins (Table S2) shows that the Ayuquila basin (5), records six species of the nematode genus *Rhabdochona* from a total of 15 species of helminths; in Durango (14) records belong to seven species in the monogenean family Dactylogyridae, six species of the monogenean genus *Gyrodactylus*, and four species of *Rhabdochona*. The Río Atoyac basin (8) has records of three species of *Rhabdochona*, two Dactylogyridae, and two species of the trematode *Paracreptotrema*, while records from Chiapas Pacífico (10) include six Dactylogyridae. Also, the absence of Phylum Acanthocephala or of records belonging to classes Monogenea and/or Cestoda or any combination of these absences in the above mentioned basins explains their position in Fig. 5, displaying a low diversity Δ+ values, and a high variation Λ+ values.

The highest helminth species richness is found in south-eastern Mexico. Contrarily, the hydrological basins with intermediate or low helminth species richness are located in northern and central areas of the country (Fig. 1). Funnels (Figs. 2 & 3) show a gradient in species richness with the south-eastern basins of Mexico (Papaloapan (20), Tabasco (21), Usumacinta (22), and Yucatán (23)) being the richest (S = 49–51) and moving from right to left towards the less rich basins in northern Mexico (Oases of Baja California (1), rivers near Chamela (3), bodies of water of the Valley of Cuatro Ciénegas (13) (S = 6–8)). Even Río Santiago (4), Río Armería-Ayuquila (5), Río Papagayo and other bodies of water of Guerrero (7), Río Atoyac (8), the rivers along the Pacific coast of Chiapas (10), and Río Bravo (11) basins rank amongst those with lower richness (S = 9–15).

**Figure 6:**
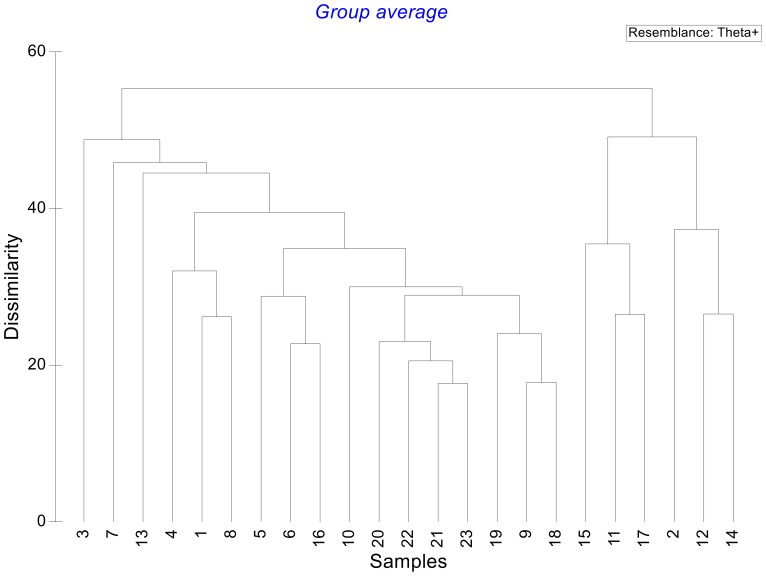
Dendrogram resulting from the dissimilarity matrix based on taxonomic distinctness, Δ^+^ for 23 Mexican hydrological basins.

The dendrogram resulting from the analysis of dissimilarity matrix based on the taxonomic distinctness (θ^+^) is presented in Fig. 6. This dendrogram shows two large clusters, not closely related as shown by the high value of dissimilarity (≈60%) between them: 1) a group of nearctic basins including the helminths from Río San Fernando (15), Río Bravo (11), Río Tuxpan (17), together with Río Yaqui (2), Río Lerma (12), and Río Nazas, Mezquital and bodies of water of Durango (14); and 2) a large neotropical group which includes the majority of the basins examined in this study. This large neotropical cluster is subdivided in turn, into smaller subgroups as follows: a truly neotropical group composed mainly by the helminths from Papaloapan (20), Tabasco (21), Usumacinta (22) and Yucatán (23) basins whose dissimilarity lies about 20%; a group including Chimalapas (19), Tehuantepec (9), and Río La Antigua (18) basins; the Chiapas Pacífico river basins (10) joins in turn at a higher dissimilarity (≈32%); the Ayuquila (5), Balsas (6), and Pánuco (16) basins on one side, and the Santiago (4), the Oases of Baja California (1), and Atoyac-Verde (8), on the other, complete this large cluster, joining this group at about (≈37%) dissimilarity. The faunas of helminths recorded from the Valley of Cuatro Ciénegas (13), Chamela (3), and Guerrero (7) basins stand alone, all with neotropical links.

**Figure 7:**
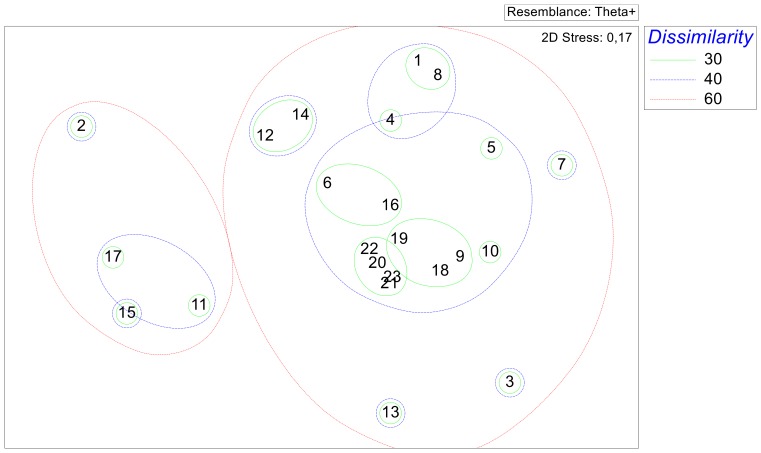
Non metric multidimensional scaling (nmMDS) ordination plot resulting from the dissimilarity matrix based on taxonomic distinctness, Δ^+^, for 23 Mexican hydrological basins.

Based on the same dissimilarity matrix, the MDS analysis (Fig. 7) shows the same trend, distinguishing a large neotropical cluster from a smaller nearctic one, well separated from one another by high dissimilarity values (>50%) between them. This analysis confirms the presence of a nuclear neotropical group consisting of the Papaloapan (20), Tabasco (21), Usumacinta (22) and Yucatán (23) basins, whose low (<30%) dissimilarity between them stresses their close taxonomic structure. To this nuclear neotropical group, other four basins are closely linked, including Chimalapas (19), Río La Antígua (18), Río Tehuantepec (9), and the rivers along the Pacific coast of Chiapas (10), the low dissimilarity between these four basins (<30%), and the close link to the nuclear group underlines the neotropical character of the helminth fauna recorded from these basins. The Río Balsas basin (6) groups with Río Pánuco (16), Río Ayuquila (5), and with Río Santiago (4) (dissimilarity ≈30%), displaying a clear neotropical affinity. MDS analysis (Fig. 6) also allows the possibility to distinguish a neotropical Pacific group of basins. Because of their orientation we can point out Oases of Baja California (1), Río Atoyac (8), Río Papagayo and other bodies of water from Guerrero (7), as neotropical basins with a Pacific affinity (Fig. 1, 6). The faunas of helminths recorded from the Valley of Cuatro Ciénegas (13), and Chamela (3) basins stand alone, with neotropical links. The nearctic group includes the Río Bravo (11), Río Tuxpan (17), and Río San Fernando and Soto La Marina (15) (dissimilarity ≈30%), plus the Río Lerma (12), and the bodies of water of Durango (14), together with Río Yaqui (2) (dissimilarity ≈50%).

**Figure 8:**
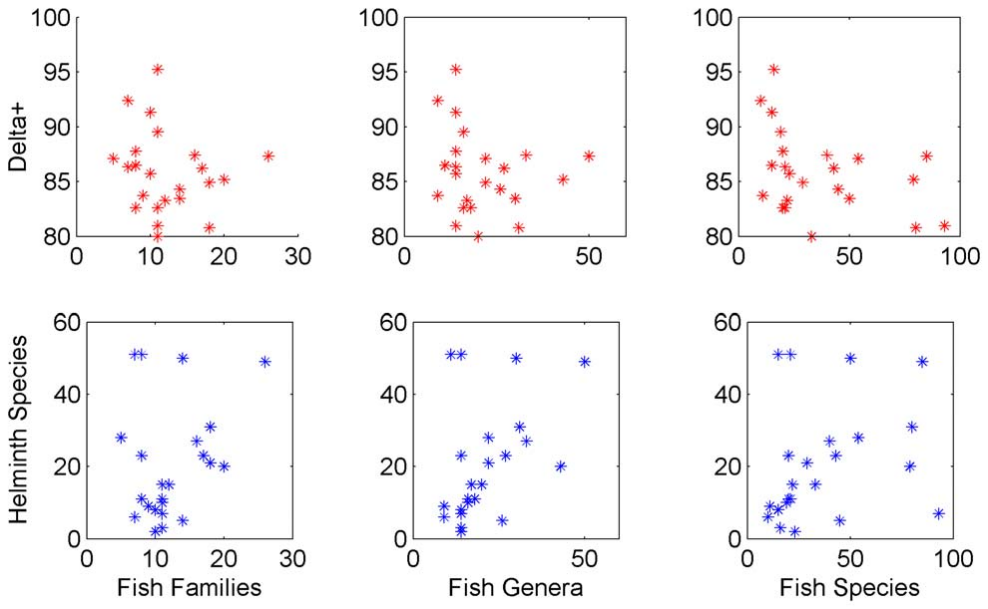
Relationships between taxonomic distinctness Δ^+^ and ichthyological diversity (no. of species; no. of genera; and no. of families of freshwater fishes) along 23 Mexican hydrological basins and between species richness and ichthyological diversity (no. of species; no. of genera; and no. of families of freshwater fishes) along 23 Mexican hydrological basins.

Correlations between helminth species richness in each basin and the number of fish species (r = 0.25, p = 0.23), the number of genera of fish (r = 0.42, p = 0.04), or the number of families of fish (r = 0.27, p = 0.2) (Fig. 7) were non-significant, except correlation 3 (Fish genera vs. Helminth species) which showed a significant relationship. Also, no significant relationship was found between diversity as measured by Δ^+^ and the number of fish species (r = −0.16, p = 0.47), the number of genera of fish (r = −0.17, p = 0.42), and the number of families of fish (r = −0.4, p = 0.06) (Fig. 8).

**Figure 9:**
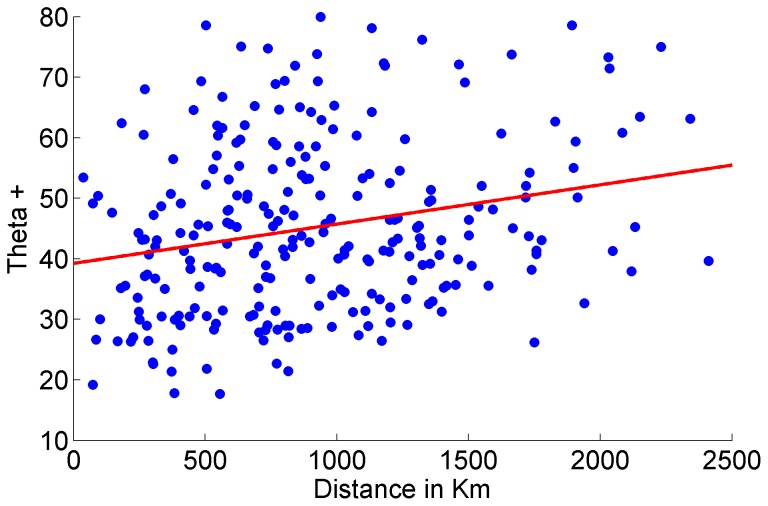
Taxonomic distinctness, Δ^+^, plotted against distance for all pairwise comparisons between drainage basins.

Finally, fig. 9 displays the relationship between Δ+ as a function of distance between all pairs of basins. The linear fit shows a positive relationship between taxonomic distinctness and distance, this means that the dissimilarity between the helminth faunas of the basins increases with the increasing distance between them.

## Discussion

This work helps clarify the evolutionary relationships amongst helminth species coexisting in an assemblage, casting a light on the diversification of parasites of freshwater fishes in Mexico. Our work allows to point out that the evolution of the fauna of helminth parasites in Mexico is mostly dominated by independent host colonization events [10], [19], such that intra – host speciation could be a minor factor explaining the origin of this diversity. The power law relationship between the number of genera and the number of species shows that most of the genera are represented monotypically in the helminth fauna of the Mexican basins; only 18% of the 84 genera bear more than three species and very few families of helminths are represented by more than one or two genera.

The present work also confirms the already described patterns of taxonomical structure of helminth fauna of freshwater fishes of Mexico, as early described by Salgado-Maldonado et al. [28], [29]. A dominance by the number of species of trematodes and nematodes is evident for all the fauna, for the helminths from each basin, and for each family of fishes [see [2] and papers cited herein]. Because of their frequency, wide distribution, and richness of species in Mexican freshwater fishes, Monogeneans are a very important taxonomical group of helminths; however, they are still insufficiently studied. Cestodes and acanthocephalans, for their part, are the less numerous groups of helminths, nevertheless, they play an important role in the diversity of each basin. If trends about variation of diversity amongst Mexican basins described in the present work are confirmed, then this means that some taxonomical groups of helminths, families and genera, would have experienced more diversification in certain basins than in others; and also, that some basins would not be colonised by certain phyla or classes of helminths.

This paper shows a clear separation between the helminth faunas of northern – nearctic and southern – neotropical components in Mexican continental waters, suggesting the availability of two distinct taxonomic pools in Mexican drainage basins. High values of Δ^+^ evidence the strong dissimilarity between neotropical and nearctic groups; values of Δ^+^ range from a minimum when all species belong to the same genus and a maximum when all species belong to different phyla [10]. Our analysis showed that populations of fishes from certain basins do not accumulate as many parasite species as others, and that fish from the nearctic region sample from a different pool of parasite species than those from the neotropical region. The size of the available pool of parasite species between the neotropical and the nearctic regions must be different; this being a constraint on the number of parasite species a fish host can acquire over time. The results and interpretations in this study are also supported by a previous study that used species richness to describe the distribution patterns of adult helminth diversity [30].

The different origin of the ichthyological faunas might explain the differences in taxonomic structure of parasite faunas. Poulin and Mouillot mention that closely related host species are likely to be parasitized by taxonomically related parasite species, because these were inherited from recent common ancestors [10]. The spatial distribution of parasites must necessarily be closely coupled with that of their hosts and therefore, the neotropical fish migrating towards Mexico [4] represent a very distinct source of parasites than the nearctic fishes and their parasites.

The present analysis confirms our previous findings [5] about patterns of distribution of helminth parasites of freshwater fishes of Mexico. The grouping of Neotropical, Nearctic and Pacific Mexican drainage basins as indicated by the helminth fauna they contain is well supported by the present findings. A closer look at the taxonomic structure and composition of the helminth faunas of Mexican hydrological basins confirms relationships between the Papaloapan (20), Tabasco (21), Chiapas (including both large river basins Grijalva and Usumacinta) (22), and Yucatán Peninsula (23) basins. This gives additional support to the concept of the Usumacinta Province as proposed by Miller [31], a concept already discussed for parasites by Vidal-Martínez and Kennedy [32], Aguilar-Aguilar et al. [33], and Quiroz-Martínez and Salgado-Maldonado [5]. The present analysis identifies this province as the nucleus of the neotropical helminth parasite fauna of freshwater fishes of Mexico. Poulin et al. [34] argue that a positive relationship between host and parasite species richness is generally supported and that this relationship is independent of variability among areas in terms of their size or in terms of sampling effort, and they also mention that hotspots of host diversity are generally hotspots of parasite diversity too [34]. The Tabasco, Papaloapan, Chiapas-Usumacinta and Yucatan Peninsula river basins are located geographically in the Mesoamerican hotspot described by Mittermeier et al. [35].

The nearctic basins include the Río Bravo (11), Río Yaqui (2), Río San Fernando (15), Río Tuxpan (17) basins, as well as the helminth faunas from the Río Lerma (12) and from various bodies of water grouped as Durango (14). Therefore, our present analysis confirms well known patterns of distribution of the fauna in Mexico: a nearctic fauna inhabiting northern locations, different from a neotropical fauna of helminth parasites of freshwater fishes inhabiting the south – south-eastern basins.

As previously proposed [5], a third set of neotropical helminths was also distinguished in present analysis: the Pacific affinities group which includes the basins located along the Pacific versant of Mexico. The records of helminths for these basins come from fishes that are distributed along the coast, entering continental bodies of water from brackish environments. The absence of these fish species from nearctic basins results in the differentiation of these basins. The present analysis confirms that the taxonomical structure of helminth assemblages is consistent enough to give support to our previous proposal [5].

Our data identifies Mexican drainage basins as unities inhabited by freshwater fishes, hosting a mixture of neotropical and nearctic species. Notoriously, the Río Balsas (6) and Durango (14), and also, the Río Lerma faunal assemblage, display an undoubtedly nearctic composition, but also show a neotropical taxonomical affinity. These findings are consistent with the Mexican Transition Zone as defined by Halffter [36], [37] an area in which Neotropical and Nearctic biotic components overlap. This zone includes South-western USA, Mexico and a large part of Central America and has been extensively studied previously [38], [39], [40], [41].

The neotropical basins of Mexico are host to a richest and more diversified helminth fauna, including more families, genera and species, compared to the less rich and less diverse helminth fauna of the nearctic basins. The fact that south-eastern basins of Mexico have higher adult helminth species richness, as opposed to northern and central basins is a well known pattern in biogeographical studies in Mexico [3], [5], [42], [43], [44], [45]. This is confirmed by the present analysis although the reasons why remain unexplored. As shown previously [5], distance decay in similarity of helminth assemblages, is an important factor that explains the observed patterns of helminth distribution. Closer basins may share hosts and parasites, leading to a highly homogeneous, but also to an enriched parasite fauna; contrary to the isolated basins, where the arrival of hosts and parasites must be a heavy constraint for the increase of the species richness. In our present results the neotropical basins of Mexico analysed here are closer together than the northern nearctic basins. The neotropical area of Mexico is a mountainous landscape, drainage basins are separated by mountain ranges, however, they are geographically closer to each other than the nearctic basins. The nearctic realm of Mexico includes not only very high mountain ranges (the Sierra Madre Oriental and the Sierra Madre Occidental) but also great desert areas (the Desierto Chihuahuense, and the Desierto Sonorense). In this area, the bodies of water lay wider apart and are isolated from one another. Such is the case of the Oases of Baja California, belonging to the Sonoran Desert, separated from the rivers near Chamela by the Pacific Ocean; similarly, the bodies of water of the Valley of Cuatro Ciénegas are surrounded by mountain ranges and extensive desert areas. Helminth richness is limited to by the possibility of colonisation of hosts carrying parasites to such remote locations, in addition to the intra – host speciation events that, as pointed out earlier in this paper, seem to be a minor factor of diversification in Mexican freshwater fishes. The present analysis confirms distance – decay as one of the important factors contributing to explain the patterns of diversity observed, with geographical distance amongst basins being a determinant of the similarity between helminth faunas [9], [34], [46].

We have previously discussed [5] the relationship between helminth richness and ichthyological complexity of Mexican drainage basins considering its geographical position along the country, proposing that ichthyological complexity is an important determinant of the richness of the helminth fauna. However, the hypothesis that helminth diversity would be explained by the ichthyological diversity of the basin received no support from the present analysis. In fact, helminth richness and Δ+ values do not correlate at all (or very lightly) to the ichthyological complexity of each basin. It seems now that helminth diversity is independent of the number of families, genera, or species of fishes inhabiting a given basin. Patterns of distribution of helminth species could be more related to the ancient geology of the basins, which explains the distribution of fish that inhabit them, rather than to the modern hydrological or limnological characteristics of each basin. These ideas could be pursued in subsequent papers.

Finally, given the relatively incomplete knowledge of the north nearctic helminth fauna of Mexico at present a caution call is in order. Indeed, freshwater fishes from nearctic basins of Mexico have only recently been studied for helminth parasites. For example, the data from the Río Bravo and main effluents derive from a single sampling campaign involving 225 fish from 13 different species. From Río Tuxpan, only data from the channel catfish (Ictalurid) are available, while from Río Yaqui data relies on a single record of two helminths. This is clearly unbalanced when compared to data from helminth parasites of the Yucatán Peninsula, sampled since 1936, and systematically studied by Moravec, Scholz and collaborators in 1995–1996; or when compared against the sampling effort undertaken by Pineda-López and collaborators when studying the helminths of freshwater fishes from Tabasco, since the early 1980’s (see references in [2]).

The theoretical model of the regional taxonomic composition of the helminth parasites of freshwater fishes of Mexico, suggests a relatively thorough knowledge of species in each basin, because most Δ+, and Λ+ values fall within 95% confidence funnels, therefore, the Mexican basins are as diverse as could be expected; this means that most basins are well sampled. However, even in relatively well sampled basins, i.e. the bodies of water in Tabasco (21), our diversity analyses suggest that the taxonomic structure of the assemblages still remains far from complete (see Fig. 3). In the same way, the continuous description of new species, named and described based on morphological taxonomy [47], [48], [49], [50] points out that even in the best studied drainage basins and fish families, the inventories are far from complete. Particularly, the study of Mexican monogeneans of freshwater fishes remains a challenge for taxonomists.

## Supporting Information

Table S1 Most speciose genera of helminths parasites of freshwater fishes of Mexico.
(DOCX)

Table S2 Taxonomic structure of the helminth parasites of freshwater fishes of Mexico. The number of families, genera and species of each class/phylum is shown under each drainage basin.
(DOCX)
