# Supplementary material for: Taxonomic Distinctness and Richness of Helminth Parasite Assemblages of Freshwater Fishes in Mexican Hydrological Basins
Source: PLoS One. 2013 Sep 27;8(9):e74419. doi: 10.1371/journal.pone.0074419 (PMC3785472; doi:10.1371/journal.pone.0074419)
Supplement: Table S1 — Most speciose genera of helminths parasites of freshwater fishes of Mexico. (DOCX) [file pone.0074419.s001.docx]

Table S1 The most speciose genera of helminths parasites of freshwater fishes of Mexico

| **Genera** | **Phylum or Class: Family** | **Number of species** |
| --- | --- | --- |
| *Rhabdochona* | Nematoda: Rhabdochonidae | 12 |
| *Gyrodactylus* | Monogenea: Gyrodactylidae | 13 (9 not yet determined) |
| *Spinitectus* | Nematoda: Cystidicolidae | 6 |
| *Procamallanus* | Nematoda: Camallanidae | 5 |
| *Neoechinorhynchus* | Acanthocephala: Neoechinorhynchidae | 5 |
| *Proteocephalus* | Cestoda: Proteocephalidae | 5 |
| *Sciadicleithrum* | Monogenea: Dactylogyridae | 5 |
| *Salsuginus* | Monogenea: Dactylogyridae | 4 |
| *Saccocoelioides* | Trematoda: Haploporidae | 4 |
| *Phyllodistomum* | Trematoda: Gorgoderidae | 4 (2 not yet described) |
| *Genarchella* | Trematoda: Derogenidae | 3 |
| *Paracreptotrea* | Trematoda: Allocreadiidae | 3 |
| *Cichlidogyrus* | Monogenea: Dactylogyridae | 3 (all introduced) |
| *Cucullanus* | Nematoda: Cucullanidae | 3 |
| *Pseudocapillaria* | Nematoda: Capillariidae | 3 (1 introduced) |
