# Supplementary material for: Taxonomic Distinctness and Richness of Helminth Parasite Assemblages of Freshwater Fishes in Mexican Hydrological Basins
Source: PLoS One. 2013 Sep 27;8(9):e74419. doi: 10.1371/journal.pone.0074419 (PMC3785472; doi:10.1371/journal.pone.0074419)
Supplement: Table S2 — Taxonomic structure of the helminth parasites of freshwater fishes of Mexico. The number of families, genera and species of each class/phylum is shown under each drainage basin. (DOCX) [file pone.0074419.s002.docx]

Table S2 Taxonomic structure of the helminth parasites of freshwater fishes of Mexico. The number of families, genera and species of each class/phylum is shown under each drainage basin. Codes used to identify each basin: 1, oases of Baja California Sur; 2, Río Yaqui; 3, rivers near Chamela, Jalisco; 4, Río Santiago; 5, Río Armería-Ayuquila; 6, Río Balsas; 7, bodies of water in Guerrero state, including Río Papagayo; 8, Río Atoyac; 9, Río Tehuantepec; 10, rivers along the south Pacific coast of Chiapas; 11, Río Bravo; 12, Río Lerma; 13, bodies of water of the Valley of Cuatro Ciénegas; 14, Río Mezquital, Río Nazas and springs of Durango state; 15, Río San Fernando, Río Soto La Marina and other bodies of water in Tamaulipas; 16, Río Pánuco; 17, Río Tuxpan; 18, Río La Antigua; 19, bodies of water of Los Chimalapas; 20, Río Papaloapan; 21, bodies of water in coastal plain of Tabasco; 22, basins of Río Usumacinta and Río Grijalva, Chiapas; 23, bodies of water of the Yucatán Península.

|  | **BASINS** | | | | | | | | | | | | | | | | | | | | | | |  |
| --- | --- | --- | --- | --- | --- | --- | --- | --- | --- | --- | --- | --- | --- | --- | --- | --- | --- | --- | --- | --- | --- | --- | --- | --- |
|  | **1** | **2** | **3** | **4** | **5** | **6** | **7** | **8** | **9** | **10** | **11** | **12** | **13** | **14** | **15** | **16** | **17** | **18** | **19** | **20** | **21** | **22** | **23** | **TOTALS** |
| Trematoda |  |  |  |  |  |  |  |  |  |  |  |  |  |  |  |  |  |  |  |  |  |  |  |  |
| Families | 1 | 1 | 1 | 2 | 4 | 4 | 4 | 1 | 6 | 3 | 5 | 3 | 5 | 4 | 1 | 5 | 2 | 6 | 5 | 8 | 8 | 8 | 7 | 9 |
| Genera | 1 | 1 | 1 | 2 | 5 | 5 | 5 | 1 | 7 | 3 | 6 | 5 | 5 | 6 | 1 | 6 | 2 | 8 | 7 | 11 | 16 | 16 | 11 | 28 |
| Species | 1 | 1 | 1 | 2 | 5 | 5 | 7 | 2 | 8 | 3 | 6 | 7 | 5 | 7 | 1 | 6 | 2 | 9 | 8 | 14 | 18 | 18 | 15 | 48 |
| Monogenea |  |  |  |  |  |  |  |  |  |  |  |  |  |  |  |  |  |  |  |  |  |  |  |  |
| Families | 2 |  | 1 | 2 |  | 2 |  | 2 | 2 | 2 | 1 | 3 | 1 | 2 |  | 1 |  | 2 | 2 | 2 | 3 | 2 | 2 | 5 |
| Genera | 2 |  | 1 | 2 |  | 5 |  | 3 | 5 | 7 | 1 | 3 | 1 | 7 |  | 2 |  | 5 | 6 | 8 | 12 | 4 | 11 | 20 |
| Species | 2 |  | 1 | 2 |  | 5 |  | 3 | 6 | 7 | 1 | 4 | 1 | 14 |  | 3 |  | 5 | 7 | 13 | 16 | 10 | 16 | 44 |
| Cestoda |  |  |  |  |  |  |  |  |  |  |  |  |  |  |  |  |  |  |  |  |  |  |  |  |
| Families | 1 | 1 | 1 | 1 | 1 | 2 |  | 1 |  |  | 1 | 2 |  | 3 | 1 | 1 | 1 |  | 1 | 3 | 3 | 2 | 2 | 4 |
| Genera | 1 | 1 | 1 | 1 | 1 | 3 |  | 1 |  |  | 2 | 4 |  | 4 | 1 | 1 | 2 |  | 1 | 4 | 4 | 2 | 2 | 7 |
| Species | 1 | 1 | 1 | 1 | 1 | 4 |  | 1 |  |  | 2 | 4 |  | 4 | 1 | 1 | 2 |  | 1 | 4 | 5 | 2 | 3 | 14 |
| Acanthocephala |  |  |  |  |  |  |  |  |  |  |  |  |  |  |  |  |  |  |  |  |  |  |  |  |
| Families | 1 |  | 1 | 1 |  | 1 | 1 |  |  | 1 |  |  |  | 2 |  | 1 |  |  | 1 | 1 | 1 | 1 | 2 | 4 |
| Genera | 1 |  | 1 | 1 |  | 1 | 1 |  |  | 1 |  |  |  | 2 |  | 1 |  |  | 1 | 2 | 2 | 3 | 3 | 6 |
| Species | 1 |  | 1 | 1 |  | 1 | 1 |  |  | 1 |  |  |  | 2 |  | 2 |  |  | 1 | 3 | 3 | 3 | 3 | 10 |
| Nematoda |  |  |  |  |  |  |  |  |  |  |  |  |  |  |  |  |  |  |  |  |  |  |  |  |
| Families | 1 |  | 2 | 3 | 4 | 7 | 2 | 1 | 5 | 3 | 2 | 4 | 1 | 1 | 1 | 4 | 1 | 5 | 8 | 8 | 7 | 8 | 8 | 12 |
| Genera | 1 |  | 3 | 3 | 4 | 7 | 2 | 1 | 5 | 3 | 2 | 4 | 1 | 1 | 1 | 6 | 1 | 6 | 9 | 10 | 7 | 10 | 12 | 23 |
| Species | 1 |  | 4 | 4 | 9 | 13 | 3 | 3 | 7 | 4 | 2 | 5 | 1 | 4 | 1 | 11 | 1 | 9 | 10 | 15 | 8 | 18 | 14 | 54 |
| **TOTALS** |  |  |  |  |  |  |  |  |  |  |  |  |  |  |  |  |  |  |  |  |  |  |  |  |
